# Effects of spinal–epidural anesthesia combined with intravenous etomidate on adrenocortical and immune stress in elderly patients undergoing anorectal surgery: A retrospective analysis

**DOI:** 10.17305/bb.2024.10759

**Published:** 2024-08-03

**Authors:** Yangyi Li, Jiangyan Wu, Chengbo Chen, Changsheng Su

**Affiliations:** 1 Department of Anesthesiology, Quanzhou First Hospital Affiliated to Fujian Medical University, Fujian, China; 2 Department of Cardiology, Quanzhou First Hospital Affiliated to Fujian Medical University, Fujian, China

**Keywords:** Combined spinal–epidural anesthesia (CSEA), etomidate, anorectal surgery

## Abstract

The management of anesthesia in elderly patients undergoing surgery presents unique challenges, particularly in mitigating stress responses and ensuring stability. Etomidate may alleviate adrenocortical and immune stress. This study aims to investigate the anesthetic effects of combined spinal–epidural anesthesia (CSEA) supplemented with etomidate during anorectal surgery in elderly patients. The medical records of 49 cases treated with CSEA and etomidate (ETO group) and 48 cases treated with CSEA alone (control group) were reviewed and analyzed. All patients received ropivacaine hydrochloride for anesthesia, with the ETO group additionally receiving an infusion of etomidate for sedation. Parameters, such as arterial blood gas, visual analog scale (VAS), Ramsay sedation scale (RSS), serum cortisol and norepinephrine levels, proinflammatory cytokines, and lymphocyte ratios, were assessed at different time points. Compared to the control group, the ETO group showed increased mean arterial pressure (MAP), decreased heart rate (HR), and elevated arterial SpO_2_ 30 min after anesthesia. The ETO group also had higher RSS scores, lower VAS scores, and reduced serum cortisol and norepinephrine levels. Additionally, decreased levels of proinflammatory cytokines, such as interleukin (IL)-6, tumor necrosis factor (TNF)-α, and IL-8, were observed, along with an increase in the regulatory cytokine IL-10. An increased proportion of CD4+ T cells and a higher CD4/CD8 ratio were also noted. This study demonstrates the benefits of using etomidate to mitigate adrenocortical and immune stress in elderly patients undergoing anorectal surgery.

## Introduction

Anorectal surgery is recommended for proctologic disorders affecting the anus, rectum, and pelvic floor [1, 2]. Proctologic disorders are highly prevalent (4%–5%) and cause significant morbidity and mortality [3, 4]. Elderly patients are more likely to have concomitant diseases, such as pulmonary and cardiovascular comorbidities, diabetes mellitus, and hyperlipidemia, which can increase the perioperative risk and the need for more careful anesthesia management. Combined spinal–epidural anesthesia (CSEA) is recommended for elderly patients during anorectal surgery to alleviate the stress induced by the operation. CSEA is a relatively new regional anesthesia technique that includes an initial subarachnoid injection, followed by epidural catheter placement, and subsequent epidural administration to achieve a controlled anesthesia level and selective segmental block [5–7]. Etomidate has the unique characteristics of hemodynamic stability and minimal respiratory depression among the rapid-onset induction agents, providing a wider margin of safety than barbiturates or propofol [8]. In general anesthesia induction, etomidate has been utilized for patients undergoing cardiac surgery or with poor cardiac function [9]. Additionally, etomidate anesthesia shows little influence on the immune functions of patients with infectious shock and has a low incidence of adverse reactions [10]. Theoretically, etomidate can interact with adrenal steroidogenesis enzymes, specifically steroid 11-beta-hydroxylase, which converts 11-deoxycorticosterone to corticosterone and 11-deoxycortisol to cortisol. A meta-analysis suggests that intensivists should anticipate the need for glucocorticoid supplementation after etomidate in patients with severe critical illness and those with acute deterioration of vital signs [11]. These findings indicate that the supplementary use of etomidate might have the potential to alleviate adrenocortical and immune stress during anorectal surgery in elderly patients. The current study aims to evaluate the anesthetic effects of CSEA supplemented with etomidate during anorectal surgery in elderly patients.

## Methods and materials

### Patient samples

Elderly patients who underwent anorectal surgery at Quanzhou First Hospital, affiliated with Fujian Medical University, from February 1, 2019, to February 1, 2020, were reviewed and retrospectively analyzed in this study. Two groups of patients were analyzed: one group received traditional CSEA treatment (control group), and the other group received etomidate combined with CSEA (ETO group). The sample size was calculated using established statistical power analysis. Age matching and disease type matching were used as baseline information between the different groups. The visual analog scale (VAS) and Ramsay sedation scale (RSS) were used as primary outcome indicators. Secondary outcome indicators included arterial pressure, heart rate (HR), blood oxygen saturation, stress indicators, and immune function indicators. Differences in VAS between the two groups were divided by the standard deviation (SD) to determine the standardized effect size, and 5% was used as the significance level in the Mann–Whitney test with 90% power.

Inclusion criteria: Age > 60 years old; who met the diagnostic criteria for anorectal diseases and surgical indications; who met the American Society of Anesthesiologists (ASA) physical status classification (I–III).

Exclusion criteria: Contraindications to anesthesia; abnormal coagulation function; severe cardiovascular and cerebrovascular diseases; abnormal immune function; organ dysfunction or malignant tumor; cumulative time of blood pressure < 90/60 mmHg during the operation greater than 15 min, or cumulative time of blood pressure > 160/110 mmHg greater than 15 min; shock and bradycardia during operation; missing statistics data. The research was designed according to the Specification for Reporting Observational Studies (STROBE), and it was also approved by the Ethical Committee of Quanzhou First Hospital affiliated with Fujian Medical University.

### Anesthesia procedure

In the central operating room, the Datex-Ohmeda S/5 Monitor was used to monitor SpO_2_, non-invasive blood pressure, and electrocardiogram. Oxygen inhalation (oxygen flow rate of 2 l/min) was performed for 5 min, epidural puncture was performed in the L2–L4 intervertebral space, and then lumbar puncture was carried out with puncture needles. After confirming cerebrospinal fluid flow, 1 mL of ropivacaine hydrochloride (100 mg/10 mL, Zhuhai Rundu Pharmaceutical Co., Ltd., China) and 1 mL of glucose (10 g/100 mL) were infused for 10 s. After withdrawing the needles, tubes were inserted in the epidural space to facilitate further injections. The lateral decubitus position was adopted to adjust the anesthesia level.

In the ETO group, 0.3 mg/kg/h etomidate (Guangzhou Baiyunshan Mingxing Pharmaceutical Co., Ltd., China) was slowly injected intravenously before the skin incision. After the conjunctival reflex disappeared, the patients were given 0.1 mg/kg/h etomidate intravenously. Time course (before the anesthesia, T0; 30 min after the anesthesia, T1; after the operation, T2; 1 h after the operation, T3; 12 h after the operation, T4; 24 h after the operation, T5; 72 h after the operation, T6) analysis of mean arterial pressure (MAP), HR, and arterial SpO_2_ were monitored. The effectiveness of spinal anesthesia for anorectal surgery was defined as the complete disappearance of surgical pain, good relaxation of the abdominal muscles, and successful completion of the surgery.

Postoperatively, a patient-controlled analgesia pump was connected to the epidural catheter. The medication dosage was controlled according to the patients’ pain condition. The loading dose was 5 mL. The background infusion rate was 2 mL/h. The patient-controlled bolus dose was 0.5 mL per activation, with a lockout interval of 15 min. The analgesic mixture consisted of morphine 4 mg and ropivacaine 100 mg diluted with saline to 100 mL. Both groups used identical postoperative analgesia pump conditions.

### Sedative and analgesic effects

VAS with 100 mm horizontal lines to indicate the severity of pain (0–10 points) and RSS (1, anxious and agitated or restless, or both; 2, cooperative, oriented, and tranquil; 3, responds to commands only; 4, brisk response to a light glabellar tap or loud auditory stimulus; 5, sluggish response; 6, no response) were used to assess analgesic and sedative effects, as indicated in previous reports [12, 13]. The evaluation of sedative and analgesic effects was performed 1 h after the operation, 24 h after the operation, and 72 h after the operation.

### Adrenocortical stress assessment

Blood samples were collected in BD Vacutainer^®^ SST™ Venous Blood Collection Tubes from the antecubital vein, aseptically through venipuncture, at the time points T0, T1, and T5. The levels of norepinephrine were detected with high-performance liquid chromatography (3H-NE, Tiangen Corp., Beijing, China). The levels of cortisol were measured by radioimmunoassay as indicated by previous reports [14, 15].

### Immune stress assessment

The interleukin (IL)-6, tumor necrosis factor (TNF)-α, IL-8, and IL-10 levels were measured at the time points T0, T1, and T5 in the serum of the patients. The peripheral blood mononuclear cells (PBMCs) EasySep Direct Human PBMC Isolation Kit (StemCell) was used to isolate PBMCs, which were further incubated with anti-CD4-Pacific Blue antibody and anti-CD8-APC-Cy7 antibody (Invitrogen, Karlsruhe, Germany) for 20 min at 4 ^∘^C in the dark. The stained cells were measured with an LSR-II flow cytometer (Becton Dickinson).

### Ethical statement

The study was approved by the Ethical Committee of Quanzhou First Hospital affiliated with Fujian Medical University. The study was performed in strict accordance with the Declaration of Helsinki, Ethical Principles for Medical Research Involving Human Subjects.

### Statistical analysis

The sample size was calculated using established statistical power analysis. Differences between each compared treatment group were divided by the SD to determine the standardized effect size, and 5% was used as the significance level. Then, the minimum required sample size was calculated. The Shapiro–Wilk test and Kolmogorov–Smirnov test were used to test the normality of data before analysis. When at least one of these tests indicated that both groups of data followed a normal distribution, they were considered to meet the normality assumption. Values were expressed as mean ± SD. *P* values were derived from the Mann–Whitney test. The chi-square test or Fisher’s exact test was used for assessing the distribution of observations or phenomena between different groups. The significance level was set at a *P* value less than 0.05. All statistical analyses were performed using GraphPad Prism (GraphPad Software, Inc.).

## Results

### Comparison of baseline data

A total of 49 cases in the ETO group and 48 cases in the control group were included in this study. The control group was matched with the ETO group for gender, age, ASA scale, surgical specialty, and complicating diseases (Table 1). The postoperative recovery time in the ETO group was 6.8 ± 2.1 min, while in the control group, it was 7.2 ± 2.6 min. There was no significant difference in postoperative recovery time between the two groups (*P* ═ 0.105). It is worth noting that there were no significant differences in postoperative nausea and vomiting (PONV), inflammation, or hospital length of stay (LOS) between the two groups.

**Table 1:** Demographic and clinical characteristics of the study participants

**Characteristics**	**Study group**		***P***
	**Control (*n* ═ 48)**	**ETO (*n* ═ 49)**	
*Gender*			
Male	22 (45.8%)	25 (51.1%)	0.686
Female	26 (54.2%)	24 (48.9%)	
Age (years)	69.4 ± 4.8	71.2 ± 5.1	0.193
*ASA scale*			
I	15 (31.3%)	13 (26.5%)	0.542
II	23 (47.9%)	21 (42.9%)	
III	10 (20.8%)	15 (30.6%)	
*Surgical specialty*			
Mixed hemorrhoid	16 (33.3%)	14 (28.6%)	0.909
Internal hemorrhoids	9 (18.8%)	11 (22.4%)	
External hemorrhoid	6 (12.5%)	5 (10.2%)	
Low anal fistula	7 (14.6%)	10 (20.4%)	
High anal fistula	5 (10.4%)	6 (12.3%)	
Perianal abscess	5 (10.4%)	3 (6.1%)	
*Complicating disease*			
Hypertension	15 (31.3%)	13 (26.5%)	0.659
Diabetes mellitus	8 (16.7%)	5 (10.2%)	0.387
Hyperlipidaemia	11 (22.9%)	14 (28.6%)	0.644
COPD	6 (12.5%)	3 (6.1%)	0.317
PONV	3 (6.25%)	2 (4.08%)	0.678
Infections	0 (0.0 %)	0 (0.0%)	1.000
LOS (days)	8.4 ± 1.9	7.9 ± 1.7	0.133

Values were expressed as *n* (percentage, %) or mean ± SD. *P* values were derived from the Mann–Whitney test. The chi-square test or Fisher’s exact test was used to assess the distribution of observations or phenomena between different groups. ASA: American Society of Anesthesiologists; ETO: Etomidate; COPD: Chronic obstructive pulmonary disease; PONV: Postoperative nausea and vomiting; LOS: Hospital length of stay; SD: Standard deviation.

### Etomidate maintains hemodynamic stability

Abnormal MAP, HR, and SpO_2_ were observed during the operation in both groups, consistent with previous observations that surgery can affect patients’ normal oxygenation and hemodynamics [16]. A significant difference in MAP, HR, and SpO_2_ was observed 30 min after anesthesia in the ETO group compared to the control group (Table 2). In other words, ETO induced a decreased HR, higher blood oxygen saturation, and higher MAP compared to CSEA at the T1 time point. In contrast, no significant differences in HR, blood oxygen saturation, or MAP were observed at the T2 and T5 time points. These findings indicate that the early stage of hemodynamic stability could be attributed to the supplementary administration of etomidate.

### Etomidate induces sedative and analgesic effect

**Figure 1.**
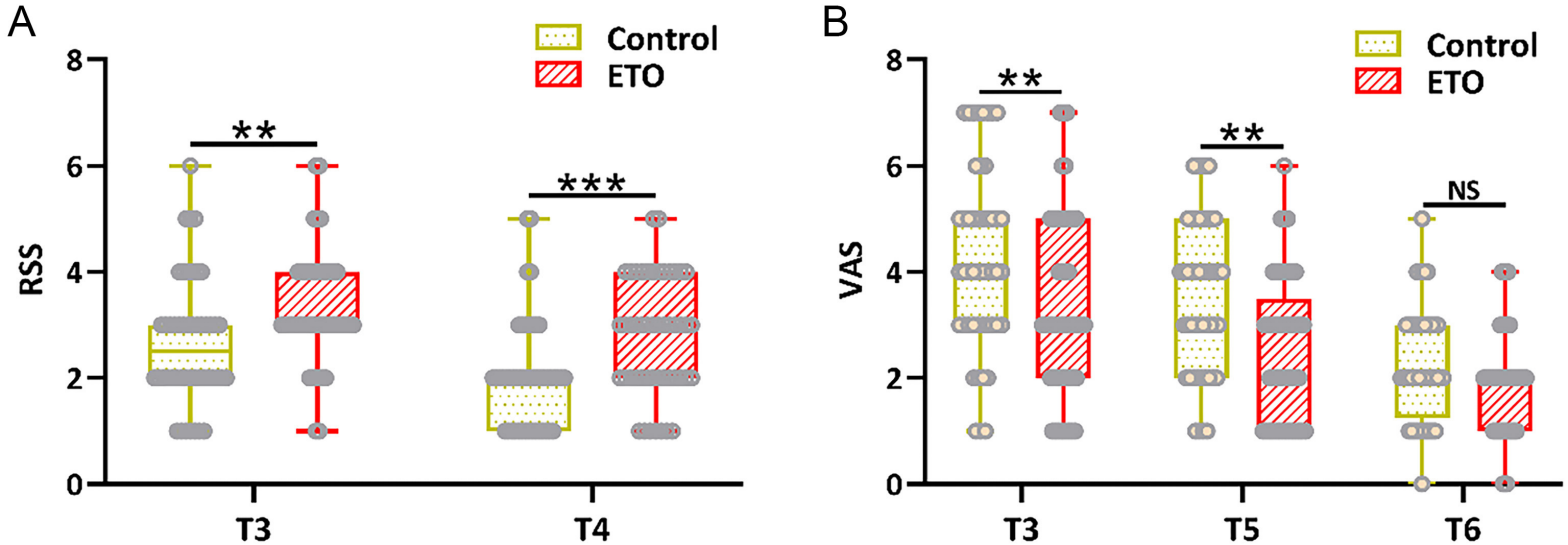
(A) Comparison of RSS scores between the two groups 1 h after the operation (T3) and 12 h after the operation (T4); (B) Comparison of VAS scores between the two groups 1 h after the operation (T3), 24 h after the operation (T5), and 72 h after the operation (T6). *n* ═ 48 for control and *n* ═ 49 for ETO. Box plot. **P < 0.01, ***P < 0.001; NS means not significant. Mann–Whitney test. VAS: Visual analog scale; RSS: Ramsay sedation scale.

An increased RSS score was observed in the ETO group at T3 and T4 compared to the control group (Figure 1A), indicating the anesthetic advantage of postoperative sedation. Additionally, ETO demonstrated an anesthetic advantage in postoperative analgesia at T3 and T5 compared to the control group (Figure 1B). These data demonstrate that the supplementary administration of etomidate can enhance sedative and analgesic effects.

**Table 2:** Comparisons of MAP, HR, and arterial SpO_2_ between the two groups

		**T0**	**T1**	**T2**	**T5**
MAP (mmHg)	Control (*n* ═ 48)	96.3 ± 6.4	79.5 ± 6.9	85.3 ± 6.8	93.9 ± 7.2
	ETO (*n* ═ 49)	95.9 ± 7.0	84.7 ± 6.5^*^	88.2 ± 7.1	94.7 ± 5.9
HR (/min)	Control (*n* ═ 48)	76.2 ± 8.1	85.3 ± 7.6	80.2 ± 9.1	78.5 ± 7.8
	ETO (*n* ═ 49)	77.4 ± 7.8	79.1 ± 8.6^*^	78.7 ± 8.4	77.1 ± 8.4
Arterial SpO_2_ (%)	Control (*n* ═ 48)	97.8 ± 3.5	91.5 ± 4.2	94.3 ± 3.8	96.9 ± 3.7
	ETO (*n* ═ 49)	97.4 ± 2.9	94.6 ± 3.3^*^	96.1 ± 3.4	97.5 ± 3.1

Values were expressed as mean ± SD. *P < 0.05 compared to control at the same time point. Mann–Whitney test. MAP: Mean arterial pressure; HR: Heart rate; SD: Standard deviation.

### Etomidate alleviates adrenocortical stress

**Figure 2.**
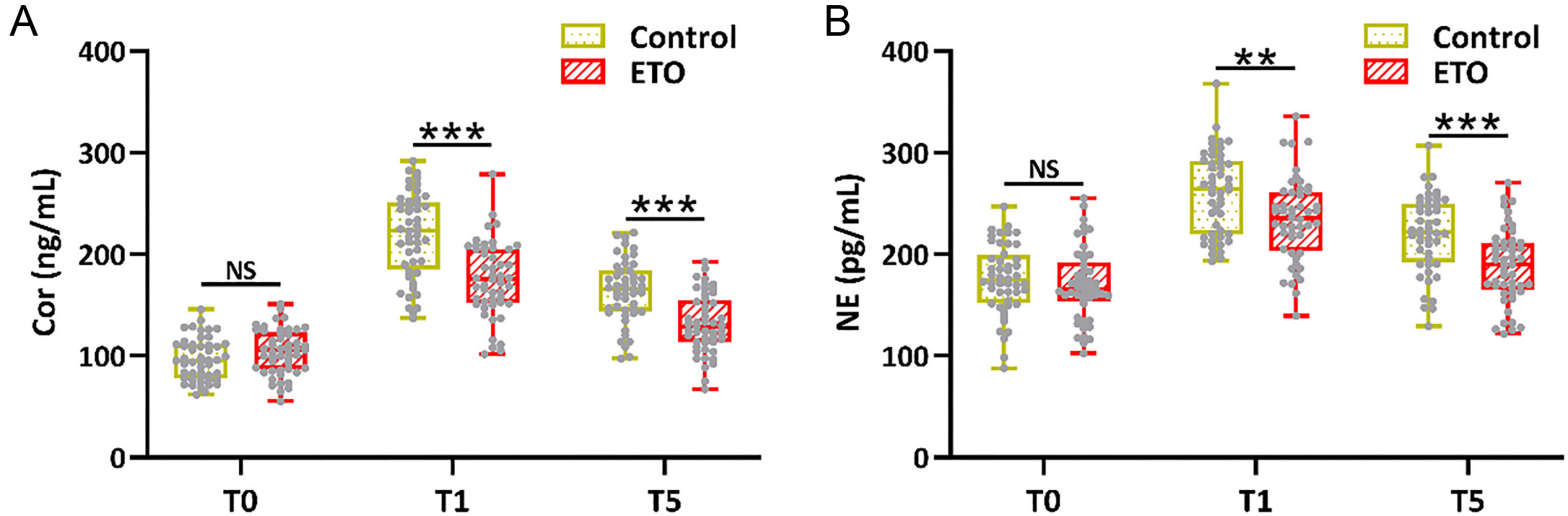
**Comparison of stress indices, including serum cortisol (A) and norepinephrine (B) between the two groups before (T0), 30 min after the start of anesthesia (T1), and 24 h after the operation (T5).** *n* ═ 48 for control and *n* ═ 49 for ETO. Box plot. **P < 0.01, ***P < 0.001; and NS means not significant. Mann–Whitney test.

As expected, cortisol and norepinephrine levels were significantly upregulated at T1 and T5 after the operation compared to T0. Conversely, diminished cortisol (Figure 2A) and norepinephrine (Figure 2B) levels were observed in the ETO group compared to the control group at T1 and T5.

### Etomidate alleviates immune stress

**Figure 3.**
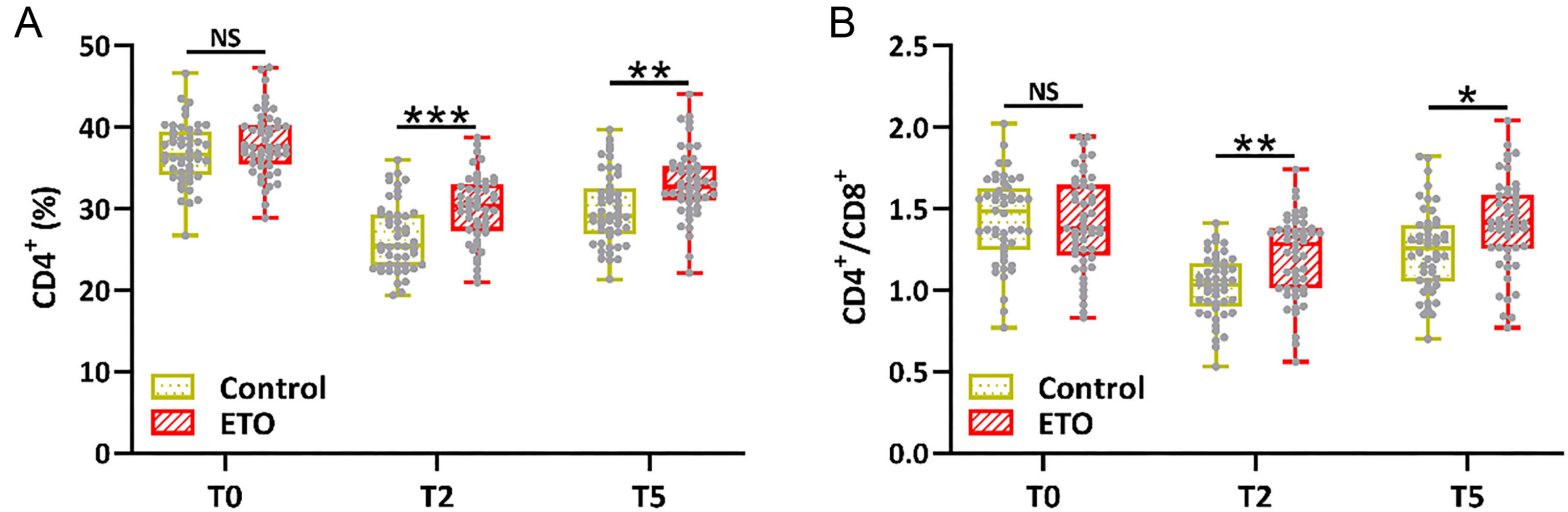
**Comparison of immune indices, including CD4 positive T cells (A) and the CD4/CD8 positive T cell ratio (B) in peripheral blood, between the two groups before anesthesia (T0), at the end of the operation (T2), and 24 h after the operation (T5).** *n* ═ 48 for control and *n* ═ 49 for ETO. Box plot. *P < 0.05, **P < 0.01, ***P < 0.001; NS means not significant. Mann–Whitney test.

**Figure 4.**
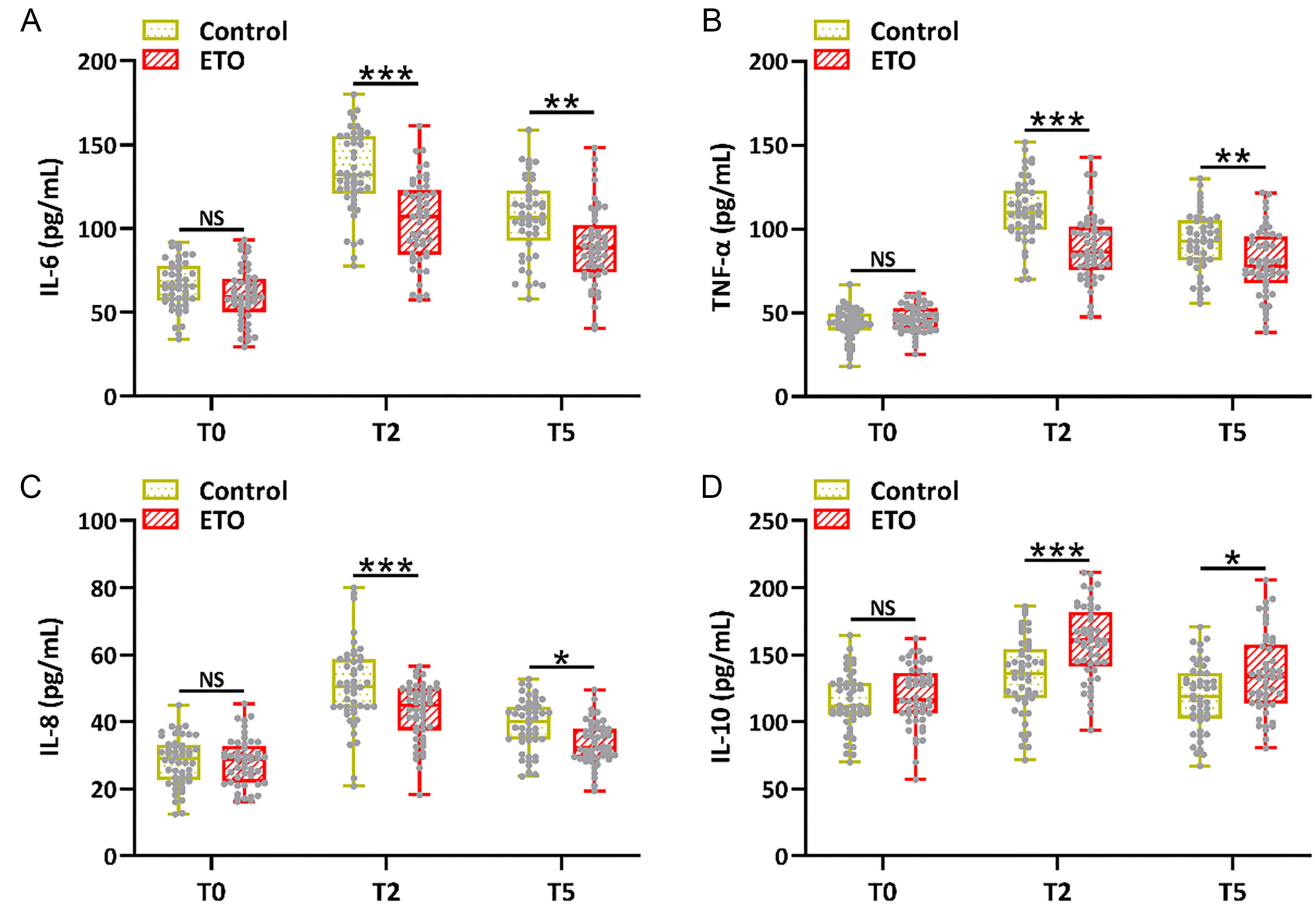
**Comparison of serum IL-6 (A), TNF-α (B), IL-8 (C), and IL-10 (D) between the two groups before anesthesia (T0), at the end of the operation (T2), and 24 h after the operation (T5).** *n* ═ 48 for control and *n* ═ 49 for ETO. Box plot. *P < 0.05, **P < 0.01, ***P < 0.001; NS means not significant. Mann–Whitney test. IL: Interleukin; TNF: Tumor necrosis factor.

The percentage of CD4+ T cells and the CD4/CD8 ratio in both groups were significantly reduced at T2 and T5 compared to the T0 stage, indicating that surgery and anesthesia can induce immune stress. As expected, an upregulated percentage of CD4+ T cells (Figure 3A) and a higher CD4/CD8 ratio (Figure 3B) were observed in the ETO group compared to the control group at T2 and T5. The increased release of proinflammatory factors caused by the operation and anesthesia was observed at T2 and T5 compared to the T0 stage. Decreased IL-6 (Figure 4A), TNF-α (Figure 4B), and IL-8 (Figure 4C) levels, along with increased IL-10 levels (Figure 4D), were observed in the ETO group compared to the control group at T2 and T5. These data suggest that supplementary etomidate may be used to alleviate the hormone and immune stress induced by surgery.

## Discussion

Anesthesia may impact adrenocortical and immune stress in elderly patients undergoing anorectal surgery [17]. This study demonstrates that CSEA supplemented with etomidate provides superior sedative and analgesic benefits compared to traditional CSEA. This improvement is accompanied by a reduction in the expression of stress hormones and the CD4/CD8 ratio. Therefore, the use of etomidate in elderly patients undergoing anorectal surgery should be considered in clinical practice. Our results indicate that CSEA with intravenous etomidate significantly reduces adrenocortical stress in elderly patients undergoing surgery. Etomidate’s unique pharmacological profile, particularly its ability to inhibit adrenal steroidogenesis, results in reduced cortisol production [18]. This suppression is critical for elderly patients who are more vulnerable to the adverse effects of elevated cortisol levels during the perioperative period. Elevated cortisol, a common response to surgical stress, can lead to complications, such as impaired wound healing, hyperglycemia, and increased susceptibility to infections [19]. By maintaining lower and more stable cortisol levels, etomidate minimizes these risks, promoting a more favorable postoperative course. Moreover, CSEA with etomidate offers comprehensive analgesia and anesthesia, further attenuating the stress response. Spinal–epidural anesthesia effectively blocks afferent pain pathways, thereby reducing the central nervous system’s perception of pain and subsequent stress response [20]. When combined with etomidate, this anesthetic approach ensures minimal hemodynamic fluctuations and reduces the overall stress burden on the patient’s body. The anal canal and perianal skin are richly innervated and sensitive to pain and damage. Tissue damage associated with anorectal surgery inevitably leads to inflammation. Uncontrolled inflammation may result in systemic inflammatory syndrome or multiple organ dysfunction [21]. Inflammation or surgical stimulation of perianal skin can cause spasms and contraction of the external anal sphincter and levator ani muscle, affecting postoperative recovery [22, 23]. Etomidate acts on the central nervous system by stimulating γ-aminobutyric acid receptors and depressing the reticular activating system, demonstrating a negligible effect on hemodynamics and stress reactions during intravenous anesthesia. Etomidate can inhibit adrenocortical stress by preventing cortisol biosynthesis, thus maintaining normal plasma cortisol levels and preventing the typical increase in response to surgery [19, 24, 25]. Surgical stress and anesthesia can significantly impact the immune system, often leading to a proinflammatory state that can complicate recovery [26]. This study found that etomidate, in conjunction with CSEA, favorably modulates the immune response. Etomidate’s ability to suppress the inflammatory response is particularly beneficial in elderly patients, who typically have diminished immune reserves and a higher propensity for postoperative complications. Etomidate has been shown to alleviate surgery and anesthesia-induced inflammatory responses during lung adenocarcinoma resection compared to propofol [27]. During surgery for lower limb fractures, etomidate can maintain serum superoxide dismutase activity and inhibit the release of inflammatory factors, reducing sedation and the occurrence of anesthesia-related complications [28, 29]. In elderly patients with rheumatic heart valve disease undergoing heart valve replacement, combined etomidate–ketamine anesthesia can stabilize perioperative electrocardiogram indicators, improve postoperative cognitive function, and reduce pain sensation compared to the ketamine group [30]. These findings suggest that etomidate use should be promoted and applied in practice with more precise analysis. Studies have shown that etomidate can reduce the release of proinflammatory cytokines and maintain a more balanced immune response during and after surgery [31–33]. This immunomodulatory effect helps prevent excessive inflammatory reactions that can lead to conditions, such as systemic inflammatory response syndrome or multiple organ dysfunction syndrome. By mitigating these inflammatory responses, etomidate contributes to a smoother recovery process and reduces the incidence of postoperative complications related to immune stress. Our results align with these findings, as etomidate further decreased inflammatory activities compared to CSEA alone. However, this study has some limitations. The dosage and rate of etomidate injection can significantly affect the incidence of myoclonus [34, 35], which may impact the patient’s breathing and lead to hypoxemia. In this study, several patients experienced myoclonus, indicating that a more precise clinical design is necessary to confirm the widespread use of CSEA supplemented with etomidate and minimize the incidence of myoclonus. Secondly, the specific mechanisms underlying the alleviation of adrenocortical and immune stress by CSEA supplemented with etomidate were not elucidated in this study. Lastly, whether CSEA supplemented with etomidate could be utilized in the general population beyond elderly patients requires further investigation.

## Conclusion

Our investigation suggests that etomidate may be beneficial for elderly patients undergoing anorectal surgery by minimizing adrenocortical stress and the immune response.

## References

[1] Tirrell TF, McNamara ER, Dickie BH (2021). Reoperative surgery in anorectal malformation patients. Transl Gastroenterol Hepatol.

[2] Chung CS.

[3] Yu SWB, Rao SSC (2014). Anorectal physiology and pathophysiology in the elderly. Clin Geriatr Med.

[4] Kreuter A (2016). Proctology—diseases of the anal region. J Dtsch Dermatol Ges.

[5] Gudaityte J, Marchertiene I, Pavalkis D (2004). Anesthesia for ambulatory anorectal surgery. Medicina (Kaunas) [Internet].

[6] Guasch E, Brogly N, Gilsanz F (2020). Combined spinal epidural for labour analgesia and caesarean section: indications and recommendations. Curr Opin Anaesthesiol.

[7] Wang G, Zhang P, Li M, Wu X, Li H (2022). effect of combined spinal-epidural anesthesia and total intravenous anesthesia on hemodynamics and pregnancy outcomes of severe preeclampsia pregnant patients undergoing cesarean section. Evid Based Complement Alternat Med.

[8] Wu J, Yao S, Wu Z, Wu Z, Chu S, Xia G (2013). A comparison of anesthetic regimens using etomidate and propofol in patients undergoing first-trimester abortions: double-blind, randomized clinical trial of safety and efficacy. Contraception.

[9] Bovill JG (2006). Intravenous anesthesia for the patient with left ventricular dysfunction. Semin Cardiothorac Vasc Anesth.

[10] Wang Y, Zhang J, Zhang SJ (2017). Effects of anesthesia using propofol and etomidate on T lymphocyte subpopulation of infectious shock patients in perioperative period. J Biol Regul Homeost Agents [Internet].

[11] Albert SG, Sitaula S (2021). Etomidate, adrenal insufficiency and mortality associated with severity of illness: a meta-analysis. J Intensive Care Med.

[12] Byrom B, Elash CA, Eremenco S, Bodart S, Muehlhausen W, Platko JV (2022). Measurement comparability of electronic and paper administration of visual analogue scales: a review of published studies. Ther Innov Regul Sci.

[13] Belar A, Arantzamendi M, Payne S, Preston N, Rijpstra M, Hasselaar J (2021). How to measure the effects and potential adverse events of palliative sedation? an integrative review. Palliat Med.

[14] Vecsei P, Penke B, Katzy R, Baek L (1972). Radioimmunological determination of plasma cortisol. Experientia.

[15] Willemsen JJ, Ross HA, Jacobs MC, Lenders JW, Thien T, Swinkels LM (1995). Highly sensitive and specific HPLC with fluorometric detection for determination of plasma epinephrine and norepinephrine applied to kinetic studies in humans. Clin Chem.

[16] Guo J-R, Guo W, Jin X-J, Yu J, Jin B-W, Xu F (2014). Effects of stellate ganglionic block on hemodynamic changes and intrapulmonary shunt in perioperative patients with esophageal cancer. Eur Rev Med Pharmacol Sci [Internet].

[17] Furlan L, Francesco PD, Costantino G, Montano N (2022). Choosing Wisely in clinical practice: embracing critical thinking, striving for safer care. J Intern Med.

[18] Pence A, McGrath M, Lee SL, Raines DE (2022). Pharmacological management of severe Cushing’s syndrome: the role of etomidate. Ther Adv Endocrinol Metab.

[19] Prete A, Yan Q, Al-Tarrah K, Akturk HK, Prokop LJ, Alahdab F (2018). The cortisol stress response induced by surgery: a systematic review and meta-analysis. Clin Endocrinol (Oxf).

[20] Klimek M, Rossaint R, van de Velde M, Heesen M (2018). Combined spinal-epidural vs. spinal anaesthesia for caesarean section: meta-analysis and trial-sequential analysis. Anaesthesia.

[21] Bautmans I, Njemini R, De Backer J, De Waele E, Mets T (2010). Surgery-induced inflammation in relation to age, muscle endurance, and self-perceived fatigue. J Gerontol A Biol Sci Med Sci.

[22] Wald A, Bharucha AE, Limketkai B, Malcolm A, Remes-Troche JM, Whitehead WE (2021). ACG clinical guidelines: management of benign anorectal disorders. Am J Gastroenterol.

[23] Wald A, Bharucha AE, Cosman BC, Whitehead WE (2014). ACG clinical guideline: management of benign anorectal disorders. Am J Gastroenterol.

[24] Bowdle TA, Knutsen LJS, Williams M.

[25] Mehta MP, Dillman JB, Sherman BM, Ghoneim MM, Lemke JH (1985). Etomidate anesthesia inhibits the cortisol response to surgical stress. Acta Anaesthesiol Scand.

[26] Ivascu R, Torsin LI, Hostiuc L, Nitipir C, Corneci D, Dutu M (2024). The surgical stress response and anesthesia: a narrative review. J Clin Med.

[27] Liu J, Dong W, Wang T, Liu L, Zhan L, Shi Y (2016). Effects of etomidate and propofol on immune function in patients with lung adenocarcinoma. Am J Transl Res.

[28] Li R, Fan L, Ma F, Cao Y, Gao J, Liu H (2017). Effect of etomidate on the oxidative stress response and levels of inflammatory factors from ischemia-reperfusion injury after tibial fracture surgery. Exp Ther Med.

[29] Ackerman RS, Luddy KA, Icard BE, Piñeiro Fernández J, Gatenby RA, Muncey AR (2021). The effects of anesthetics and perioperative medications on immune function: a narrative review. Anesth Analg.

[30] Yang L, Xie J, Hou D (2022). Effect of combined etomidate-ketamine anesthesia on perioperative electrocardiogram and postoperative cognitive dysfunction of elderly patients with rheumatic heart valve disease undergoing heart valve replacement. J Healthc Eng.

[31] Khodaei S, Wang DS, Ariza A, Syed RM, Orser BA (2023). The impact of inflammation and general anesthesia on memory and executive function in Mice. Anesth Analg.

[32] Tian X (2023). Influences of etomidate combined with propofol on cognitive function, inflammation and immunity in patients undergoing gastric cancer surgery. Cell Mol Biol (Noisy-le-grand).

[33] Valk BI, Struys M (2021). Etomidate and its analogs: a review of pharmacokinetics and pharmacodynamics. Clin Pharmacokinet.

[34] Do SH, Han SH, Park SH, Kim JH, Hwang JY, Son IS (2008). The effect of injection rate on etomidate-induced myoclonus. Korean J Anesthesiology.

[35] Doenicke AW, Roizen MF, Kugler J, Kroll H, Foss J, Ostwald P (1999). Reducing myoclonus after etomidate. Anesthesiology.

